# Obtaining district-level health estimates using geographically masked location from Demographic and Health Survey data

**DOI:** 10.1186/s12942-020-0198-4

**Published:** 2020-02-10

**Authors:** Emily Wilson, Elizabeth Hazel, Lois Park, Emily Carter, Lawrence H. Moulton, Rebecca Heidkamp, Jamie Perin

**Affiliations:** Johns Hopkins University Bloomberg School of Public Health, Baltimore, MD USA

**Keywords:** District, Geolocation, Disaggregation, DHS, Household surveys, Subnational

## Abstract

**Background:**

Demographic and Health Survey (DHS) data are an important source of maternal, newborn, and child health as well as nutrition information for low- and middle-income countries. However, DHSs are often unavailable at the administrative unit that is most interesting or useful for program planning. In addition, the location of DHS survey clusters are geomasked within 10 km, and prior to 2009, may have crossed district boundaries. We aim to use DHS surveyed information with these geomasked coordinates to estimate district assignments for use in health program planning and evaluation.

**Methods:**

We developed three methods to assign a district to a geomasked survey cluster in two DHS surveys from Malawi: 2000 and 2004. Method A assigns districts of origin in proportion to the likelihood that results from repeated simulated geomasking, allowing more than one possible district of origin. Method B assigns a single district of origin which contains the greatest proportion of simulated geomasked survey clusters. Method C maps the geomasked survey cluster’s location to a district polygon. We used these method assignments to estimate a selection of commonly used coverage indicators for each district. We compared the district coverage estimates, confidence intervals, and concordance correlation coefficients, by each of the methods, to those which used validated district assignments in 2004, and we looked at coverage change from 2000 to 2004.

**Results:**

The methods we tested each approximated the validated estimates in 2004 by confidence interval comparison and concordance correlation coefficient. Estimated agreement for method A was between .14 and .98, for method B the estimated agreement was between .97 and .99, and for method C the agreement ranged from .93 to .99 when compared with the validated district assignments. Therefore, we recommend the protocol which is the simplest to implement—method C—overlaying geomasked survey cluster within district polygon.

**Conclusions:**

Using geomasked survey clusters from DHSs to assign districts provided district level coverage rates similar to those using the validated surveyed locations. This method may be applied to data sources where survey cluster centroids are available and where district level estimates are needed for program implementation and evaluation in low- and middle-income settings. This method is of special interest to those using DHSs to study spatiotemporal trends as it allows for the utilization of historic DHS data where geomasking hinders the generation of reliable subnational estimates of health in areas smaller than the first-order administrative unit (ADM1).

## Background

There is potential for maternal, newborn, and child health, and nutrition (MNCH&N) programs to be informed and evaluated using household surveys [1], while health information systems are scaled-up to adequate quality in low- and middle-income countries (LMICs) [2]. The Demographic and Health Survey (DHS) program in particular provides systematic technical expertise to Ministries of Health in LMICs to design and implement household surveys for nationally representative health information [3]. Collectively, DHS represents an invaluable resource for international health organizations, researchers, and policy makers, and has been utilized in many contexts since the DHS’s inception in 1984 [4–6].

The DHS program includes the collection of standard MNCH&N coverage indicators, which are often used at the national level for accountability purposes, even though health system requirements are known to vary considerably within countries [7]. As a result, program implementation, intervention coverage, and policy decisions are increasingly important in sub-national areas [8]. In Malawi, for example, the two most recent DHSs, in 2010 and 2015–2016, were sampled to offer reliable coverage estimates at the second-order administrative unit (ADM2), or districts [9]. In most cases, however, DHSs are designed to be representative at the first-order administrative unit (ADM1), often the province or region.

### DHS geographic masking

In many surveys, DHS collects a coordinate for the centroid of the survey cluster, also referred to as the primary survey cluster defined by enumeration areas from a country’s most recent census [10]. This coordinate is geographically masked to protect the identity of those who participate. The geomasking procedure randomly geomasks the centroid location up to 10 km prior to public release in a two-step process. First, an angle, and second, a distance, are randomly chosen. In urban areas, this distance is between 0 and 2 km, while in rural areas, the distance chosen is between 0 and 5 km for 99% of survey clusters, and between 0 and 10 km for a random 1% of survey clusters [11]. Official census and DHS criteria for urban–rural distinction is country-specific and may be based on population size or infrastructure [12, 13].

This geomasking process produces an approximate location of each household while preserving privacy of survey participants. Surveyed household locations can be used to assess spatial variation in health outcomes [14], determine the predictive value of geographic factors on health [15], and link household and facility surveys [16]. In practice, even when finer resolution of surveyed locations is of interest, the geomasking protocol is often ignored, and coordinates are used as given [17]. There has been some analysis from within the DHS program to address the sensitivity in statistical applications involving spatial geomasking. In addition, the data management protocol for DHSs was updated in 2009 so that survey clusters are not geomasked across district boundaries [11]. However, for surveys prior to 2009, it is possible that the geomasking process would place the geographic coordinates of a survey cluster centroid in a district other than the one from which it was sampled, thus misidentifying the district of those who were surveyed.

Our objective, as part of the National Evaluation Platform project [18], is to use household survey data with geomasked coordinates, publically provided by DHS, to calculate indicator coverage estimates for each district by associating survey clusters inside a district, and then using household data within a district to calculate estimates and variances of coverage indicators widely used in the MNCH&N community. We chose two surveys prior to 2009, to introduce the potential that survey clusters may have been displaced across district lines. To our knowledge there has not yet been an analysis of how best to handle DHS geomasking with a statistical approach to determine the district identity of survey clusters, with the aim of identifying, summarizing, and utilizing district level MNCH&N data in program evaluation or health system planning. We tested three methods which approximate the district location of each survey cluster, in the Malawi 2000 and 2004 DHSs and make recommendations for using district level estimates and examining district-level trends in coverage based on these results.

## Methods

**Fig. 1:**
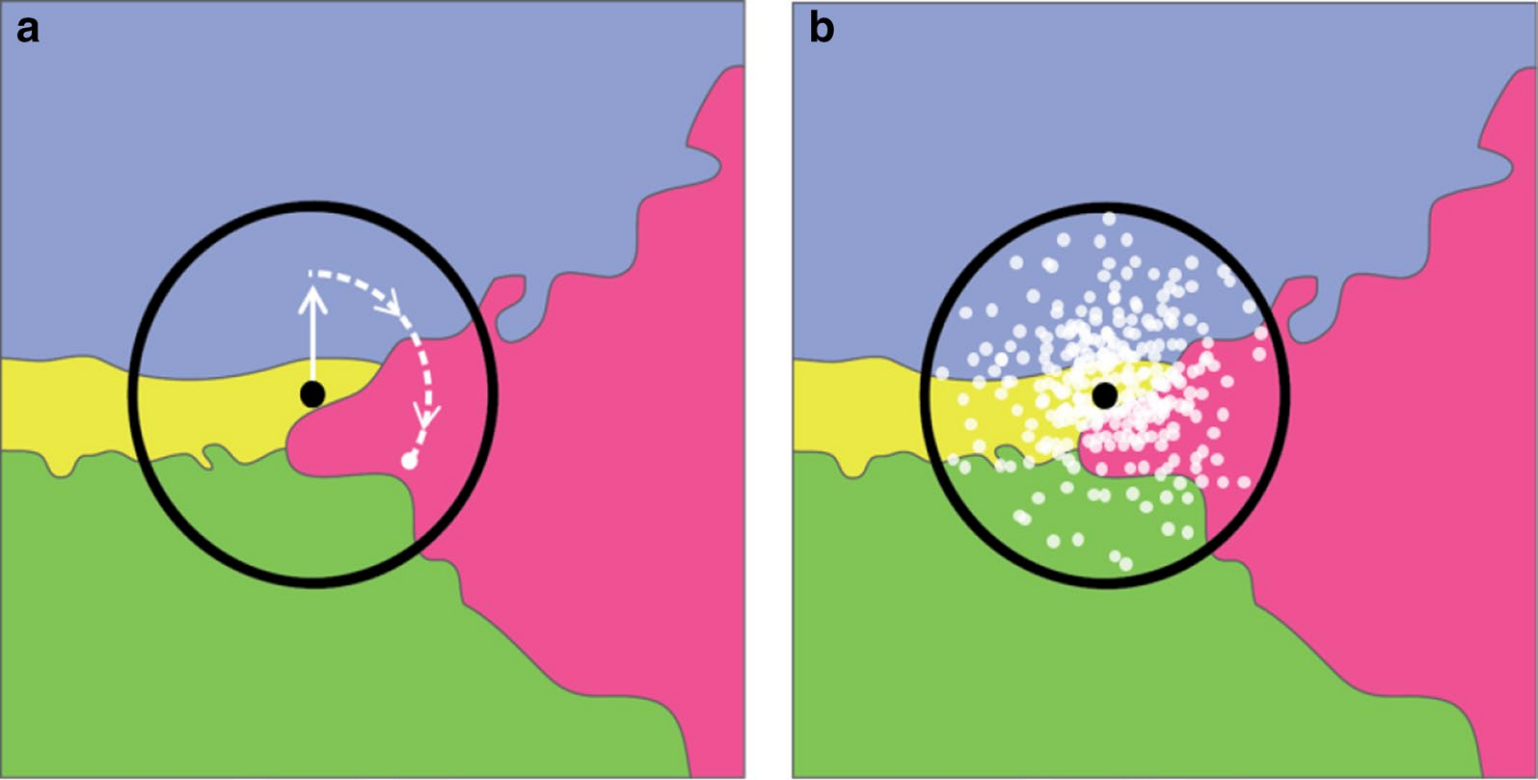
**a** The simulation process to identify possible districts of origin (green, yellow, purple, and pink areas), for a hypothetical survey cluster from a Demographic and Health Survey. The maximum displacement area with a radius of two km in an urban area or five km in a rural area (black circle) surrounds the mapped sampling unit location (black dot). First, a distance is chosen (white solid arrow), and then, an angle is chosen, (white broken arrow). **b** This process is simulated 1000 times to estimate relative likelihoods for possible districts of origin. 200 simulations (white circles) are pictured, method A assigns all four districts non-zero probability proportions: 0.13, 0.24, 0.33, 0.29 (green, yellow, blue, and pink, respectively), while method B identifies the blue district, and method C identifies the yellow district as the district of origin

We aimed to systematically estimate the area from which a survey cluster may have originated. It was not possible to recover a geomasked location, however, we specified the possible original districts, given the publicly available location of the geomasked survey cluster. Because area is increasing at distances further from the origin, the DHS geomasking process is not purely area-based, as in a dart board. A dart board would generate locations evenly distributed throughout an area, e.g., within 2 or 5 km. The DHS geomasking algorithm generates locations that are more likely to be in the area closer to the origin than the administrative boundary limit. To simulate all the locations from which a survey cluster could originate, we first selected a random distance, up to the 5 km maximum in rural areas, and 2 km maximum in urban areas, and then selected a random angle, illustrated in Fig. 1. For each survey cluster, we repeated this process 1000 times. We then used district boundaries to identify districts from which the survey cluster could possibly have been located prior to DHS geomasking. We ignored the 1% chance that a rural cluster is geomasked by more than 5 km.

We used these simulated locations in two distinct ways to estimate the district of origin. In the first method, method A, we used the respective frequencies of each simulated district, represented as a fraction of the total 1000 simulated locations for each survey cluster. For example, if simulations identified four districts as possible districts of origin (Fig. 1a), each of these districts was assigned the proportion of simulations out of 1000 that resulted from the simulation process (Fig. 1b). In method B, we selected the district with the greatest respective likelihood, out of all possible districts of origin. For method C, we selected the second order administrative unit which contained the geomasked survey cluster, as the district of origin.

### Analysis

For each of three methods, we used the resulting district assignments to estimate three coverage indicators: the proportion of households with piped water, the proportion of children under five who were moderately or severely stunted, and the proportion of infants up to 6 months old who were exclusively breastfed. We chose these measurements to cover a variety of contexts, and for variety in the expected estimate precision. The number of households, not children under 5 years, nor infants under 6 months, are fixed by survey design [19]. Therefore, all or nearly all households in this survey have information on water source, however, not all households have information on children under 5 years of age, and even fewer households have information on infants under 6 months. Thus, we expected stunting to be less precise than the piped water estimate and we expected exclusive breastfeeding to yield the least precise estimate of the three indicators.

We used the open source software R version 3.5.1 to assign districts for all three methods. For method A, we accounted for differences in probability of survey cluster selection by taking the product of the district likelihood and the survey cluster sampling weights [19]. Standard errors and confidence intervals were calculated using the Taylor linearized variance estimation [20]. Coverage estimates and their approximate standard errors were obtained using Stata 13. Source code to replicate methods and analyses is publicly available (see “Availability of data and materials”).

For the 2004 DHS survey, we also had validated district locations of each survey cluster by verbal communication from the Nation Statistical Office in Malawi. While these validated district locations are not available publicly, we were able to use them to get validated district estimates. We then compared the resulting district estimates of each method to the validated district estimates [20]. We were not able to obtain validated district locations for the 2000 DHS survey in Malawi, and so we do not include a comparison to validated estimates for 2000.

We used geomasked latitude and longitude coordinates, obtained from the geographic dataset for the Malawi 2000 and 2004 DHS surveys; additionally, we used second-order administrative boundaries in Malawi, from the Food and Agriculture Organization (FAO) of the United Nations within the Food Security for Action Programme [21], which has updated and archived all countries, and every year from 1990 to 2014. Employing these two sources of geospatial information, geomasked coordinates and administrative district boundaries, we pursued the three aforementioned methods to identify the district of origin for the survey clusters in Malawi’s 2000 and 2004 DHSs.

We included 26 districts in our analysis, out of 28 districts in Malawi at the time of the 2004 survey. We combined Neno with Mwanza in all analyses, as these districts were not separately distinguished in 2000. In addition, the island district of Likoma contained only one survey cluster in both 2000 and 2004, so we excluded Likoma from our analysis. We compared the number of survey clusters assigned to each district in three different district assignment methods as well as the validated districts. Using each method, we compared estimated coverage and confidence intervals, at the second-order administrative unit level, for three indicators against estimates based on Malawi’s National Statistics Office-confirmed validated second-order administrative units. We graphically examined estimates, described differences between estimated coverage by districts in Malawi, and assessed agreement between coverage estimates using the concordance correlation coefficient, where we had validated district assignments in 2004 [22]. This statistic ranges from − 1 to 1, where values close to 1 indicate stronger agreement [21]. We also compared the estimated district-level trends in the above described coverages for three district assignment methods A, B, and C. We assume the most reliable trend estimate is provided by the district assignment method that has the highest agreement with the validated district in their estimate from the 2004 DHS.

## Results

The number of actual survey clusters per district, as well as those estimated for methods A, B, and C, are shown for 2000 and 2004, in Table 1. In many cases, geomasked survey clusters had no district borders within 5 km of a rural survey cluster, or 2 km of an urban survey cluster, and thus were associated with only one district. In 2000, the number of survey clusters largely differed by 1 to 2 between method B and method C with the exception of: Phalombe, Nsanje, Machinga, Chitipa, Chikwawa, and Mangochi, which were consistent. In 2004, Phalombe, Ntchisi, Blantyre, Mwanza, Chitipa, and Rumphi had the same number of survey clusters although more often, the number of survey clusters differed by 1 to 3 among method B, C, and the validation method. The number of survey clusters in each district using method A is accounted for by assessing both the number of survey clusters that have a geomasked radius contained entirely within district borders, as well the partial survey clusters which resulted when geomasking simulations had more than one possible district of origin.

**Table 1:** Number of primary survey clusters per district, per method, in increasing order of district area (km^2^) in 2000 and 2004

District	Malawi 2000 DHS			Malawi 2004 DHS				District Area (km^2^)
	Method A*	Method B	Method C	Method A*	Method B	Method C	Validation	
Chiradzulu	24	14	12	18	12	11	11	767
Phalombe	12	9	9	17	10	10	10	1394
Ntchisi	9	5	7	9	7	7	7	1655
Thyolo	43	32	33	48	33	36	36	1715
Nsanje	9	8	8	12	7	8	8	1942
Blantyre	43	35	36	46	36	36	36	2012
Mulanje	49	37	36	50	33	36	36	2056
Balaka	21	12	13	19	13	11	11	2193
Salima	37	32	36	36	32	35	35	2196
Mwanza	8	4	6	9	6	6	6	2259
Zomba	47	38	37	47	37	36	36	2541
Dowa	27	21	18	23	18	16	16	3041
Karonga	36	31	7	9	5	7	7	3355
Mchinji	18	14	15	16	15	13	13	3356
Ntcheu	21	16	14	18	13	15	15	3424
Dedza	26	24	22	26	22	20	20	3624
Machinga	31	34	34	40	34	36	36	3771
Nkhata Bay	11	8	6	8	5	6	6	4071
Nkhotakota	13	8	6	14	5	8	8	4259
Chitipa	5	5	5	5	5	5	5	4288
Chikwawa	17	16	16	21	16	14	14	4755
Rumphi	7	5	4	5	4	4	4	4769
Lilongwe	41	35	33	48	33	36	36	6159
Mangochi	40	36	36	44	33	36	36	6273
Kasungu	40	35	33	41	33	36	36	7878
Mzimba	41	36	37	39	37	36	36	10,430

*Method A accounts for survey clusters per district using two columns: a survey cluster’s maximum displacement area may be entirely within a district’s boundaries, or, a survey cluster may have more than one possible district of origin, in which case partial survey clusters are counted

### Estimate variability by method

**Fig. 2:**
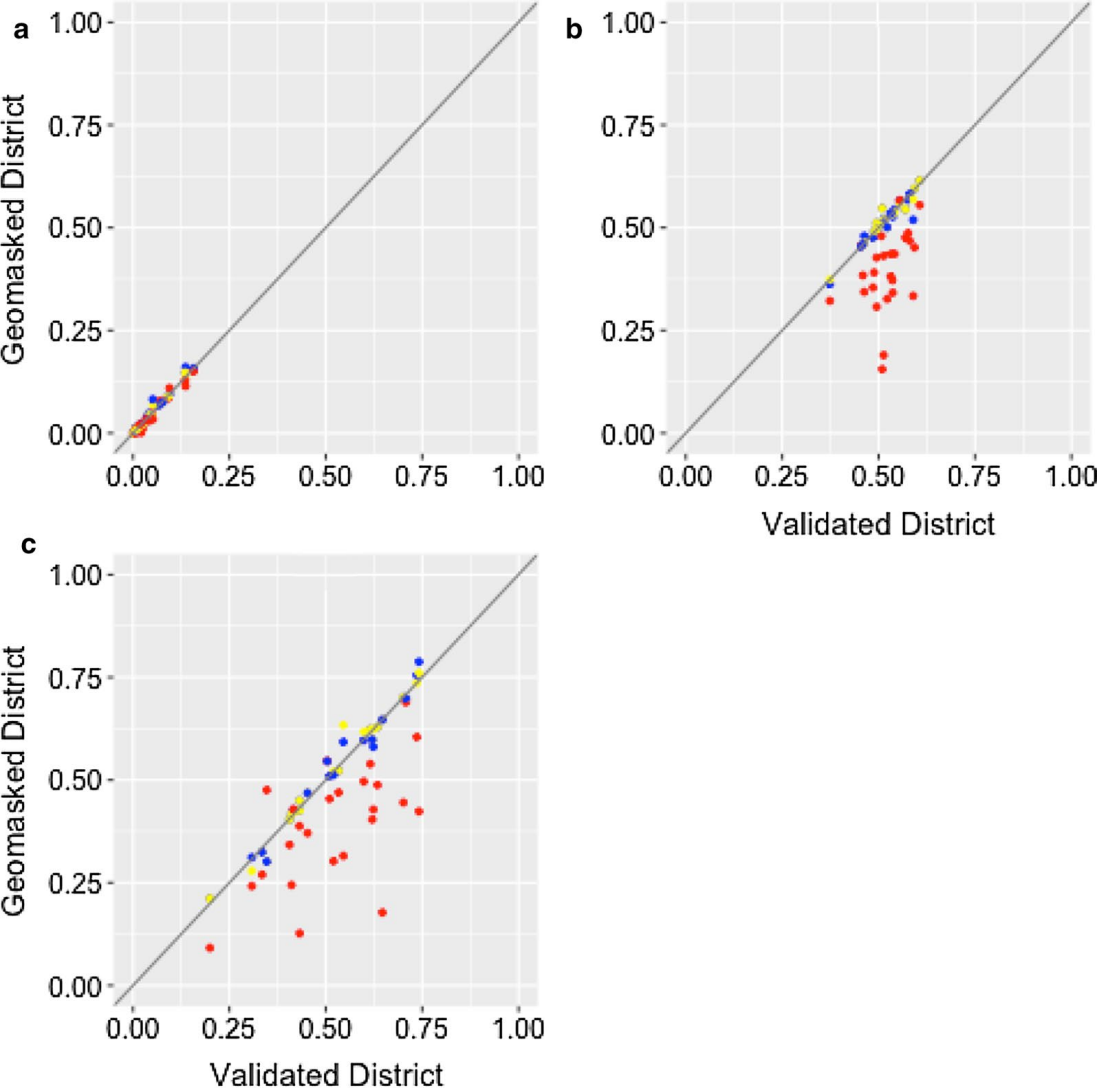
Method A (red), method B (yellow), and method C (blue) district estimates compared by indicator. Each dot represents estimated proportion coverage for a district. **a** Compares piped water by plotting approximated district estimates on the y-axis, vs. validated district estimates on the x-axis. **b** Compares stunting by plotting approximated district estimates on the y-axis, vs. validated district estimates on the x-axis. **c** Compares proportion of infants under 6 months who were exclusively breastfed by plotting approximated estimates on the y-axis vs. validated estimates on the x-axis. Dots on the diagonal line show method and validated estimate equivalence

For the 2004 survey, we plotted coverage estimates using districts approximated with methods A, B, and C against the estimates employing validated district assignments (Fig. 2) as proportions, from zero to one. Estimates appear stable for household piped water and methods B and C. We also examined approximate 95% confidence for coverage estimates, shown in Additional file 1: Tables S1–S3. All three methods produced confidence intervals that overlapped with the validation, in each district, for each of the three indicators that we tested with the exception of stunting in Chiradzulu, Lilongwe, Mulange, Mwanza, Nkhotakota, and Thyolo, for method A. The estimated proportion of households with piped water is shown in Table 2. In 24 of the 26 districts, estimates for this indicator were within ± 0.02 of the validation estimate. In the remaining two districts, Karonga and Nkhotakota, estimates did not differ among methods by more than ± 0.03. Moderate stunting estimates (Table 3) varied from the validation method by as much as ± 0.35 in Chiradzulu, ± 0.17 in Lilongwe, ± 0.22 in Mulanje, ± 0.32 Mwanza, ± 0.26 in Nkhotakota, and ± 0.19 in Thyolo, in method A. Exclusive breastfeeding among infants under 6 months (Table 4) produced estimates that varied by as much as ± 0.30 from the validation in Chiradzulu and ± 0.47 in Mwanza, ± 0.23 in Nkhotakota, and ± 0.32 in Mulanje, method A.

**Table 2:** Household piped water point estimates, by Malawi district and method, in 2000 and 2004

District	Malawi 2000 DHS			Malawi 2004 DHS				Change (2004–2000)		
	Method A (%)	Method B (%)	Method C (%)	Method A (%)	Method B (%)	Method C (%)	Validation (%)	Method A (%)	Method B (%)	Method C (%)
Balaka	5	6	6	5	4	4	5	0	− 2	− 2
Blantyre	34	35	35	15	16	16	16	− 19	− 19	− 19
Chikwawa	2	2	2	2	2	2	2	0	0	0
Chiradzulu	0	0	0	0	2	2	2	0	2	2
Chitipa	2	2	2	8	7	7	7	6	5	5
Dedza	1	1	1	1	2	2	2	0	1	1
Dowa	4	4	4	0	1	1	1	− 4	− 3	− 3
Karonga	5	4	4	11	14	16	14	6	10	12
Kasungu	4	5	5	2	2	2	2	− 2	− 3	− 3
Lilongwe	13	13	13	13	15	15	14	0	2	2
Machinga	1	2	2	0	0	0	0	− 1	− 2	− 2
Mangochi	4	4	4	3	4	4	4	− 1	0	0
Mchinji	1	2	2	1	1	1	1	0	− 1	− 1
Mulanje	2	2	2	3	4	5	4	1	2	3
Mwanza	2	3	3	11	9	9	9	9	6	6
Mzimba	9	10	8	8	9	9	9	− 1	− 1	1
Nkhata Bay	0	0	0	5	6	6	6	5	6	6
Nkhotakota	1	0	0	3	7	8	5	2	7	8
Nsanje	0	0	0	0	0	0	0	0	0	0
Ntcheu	1	1	1	2	2	2	2	1	1	1
Ntchisi	0	0	0	0	1	1	0	0	1	1
Phalombe	0	2	2	1	1	1	1	1	− 1	− 1
Rumphi	5	2	10	1	1	1	1	− 4	− 1	− 9
Salima	3	3	4	2	2	2	2	− 1	− 1	− 2
Thyolo	1	2	1	3	4	4	3	2	2	3
Zomba	5	8	8	8	8	8	8	3	0	0

**Table 3:** Moderate stunting point estimates by district and method, in 2000 and 2004

District	Malawi 2000 DHS			Malawi 2004 DHS				Change (2004–2000)		
	Method A (%)	Method B (%)	Method C (%)	Method A (%)	Method B (%)	Method C (%)	Validation (%)	Method A (%)	Method B (%)	Method C (%)
Balaka	33	56	56	48	54	54	57	15	− 2	− 2
Blantyre	41	45	45	35	48	48	49	− 6	3	3
Chikwawa	49	51	51	43	51	51	49	− 6	0	0
Chiradzulu	33	49	48	16	55	55	51	− 17	6	7
Chitipa	47	47	47	46	45	45	45	− 1	− 2	− 2
Dedza	60	66	66	56	62	62	61	− 4	− 4	− 4
Dowa	52	65	64	45	60	59	59	− 7	− 5	− 5
Karonga	36	44	44	32	37	36	37	− 4	− 7	− 8
Kasungu	44	51	51	47	58	58	58	3	7	7
Lilongwe	48	57	57	37	53	53	54	− 11	− 4	− 4
Machinga	37	49	50	39	49	49	49	2	0	− 1
Mangochi	47	54	54	43	52	52	51	− 4	− 2	− 2
Mchinji	39	71	71	49	57	57	58	10	− 14	− 14
Mulanje	42	56	56	34	53	53	54	− 8	− 3	− 3
Mwanza	35	52	52	19	51	51	51	− 16	− 1	− 1
Mzimba	45	46	50	48	51	51	51	3	5	1
Nkhata Bay	28	54	54	36	47	49	47	8	− 7	− 5
Nkhotakota	23	50	50	33	57	52	59	10	7	2
Nsanje	46	50	50	34	46	48	46	− 12	− 4	− 2
Ntcheu	48	60	60	44	54	54	54	− 4	− 6	− 6
Ntchisi	25	57	57	44	54	54	53	19	− 3	− 3
Phalombe	45	53	53	38	53	53	53	− 7	0	0
Rumphi	28	46	36	31	49	49	49	3	3	13
Salima	48	60	62	57	56	57	55	9	− 4	− 5
Thyolo	36	50	50	33	50	50	52	− 3	0	0
Zomba	40	52	52	38	46	46	46	− 2	− 6	− 6

**Table 4:** Exclusive breastfeeding point estimates, by district and method, in 2000 and 2004

District	Malawi 2000 DHS			Malawi 2004 DHS				Change (2004–2000)		
	Method A	Method B	Method C	Method A	Method B	Method C	Validation	Method A	Method B	Method C
Balaka	13	62	62	9	21	21	20	− 4	− 41	− 41
Blantyre	45	53	53	37	47	47	45	− 8	− 6	− 6
Chikwawa	51	51	51	39	45	45	43	− 12	− 6	− 6
Chiradzulu	40	45	44	13	43	43	43	− 27	− 2	− 1
Chitipa	53	53	53	45	51	51	51	− 8	− 2	− 2
Dedza	32	35	35	47	52	52	53	15	17	17
Dowa	10	16	16	24	42	42	41	14	26	26
Karonga	35	40	40	60	74	75	74	25	34	35
Kasungu	30	37	37	27	32	32	33	− 3	− 5	− 5
Lilongwe	25	33	33	49	63	63	63	24	30	30
Machinga	43	62	63	54	62	62	62	11	0	− 1
Mangochi	49	49	49	50	62	60	60	1	13	11
Mchinji	15	26	26	55	55	55	50	40	29	29
Mulanje	40	52	51	42	76	79	74	2	24	28
Mwanza	28	50	50	18	65	65	65	− 10	15	15
Mzimba	43	43	37	34	40	40	41	− 9	− 3	3
Nkhata Bay	20	41	41	43	48	48	48	23	7	7
Nkhotakota	36	46	46	32	63	59	55	− 4	17	13
Nsanje	37	47	47	43	62	58	62	6	15	11
Ntcheu	34	47	47	24	28	31	31	− 10	− 19	− 16
Ntchisi	2	6	6	48	30	30	35	46	24	24
Phalombe	49	52	52	30	52	51	52	− 19	0	− 1
Rumphi	61	64	73	45	70	70	70	− 16	6	− 3
Salima	40	42	49	43	42	43	42	3	0	− 6
Thyolo	48	58	58	40	60	60	62	− 8	2	2
Zomba	41	51	49	69	70	70	71	28	19	21

Agreement between 2004 coverage estimates and the validated district estimates was described using the concordance correlation coefficient. Strong agreement was indicated for methods B and C between estimates and validated districts, and for method A in the coverage of piped water. For piped water, estimated agreement with the validated district assignments was 0.98 (95% confidence interval .95–.99) for method A, 0.99 (.99–1.00) for method B, and 0.98 (.96–.99) for method C. For stunting among children under five, estimated agreement with the validated district assignments was 0.14 (.00–.29) for method A, 0.97 (0.94–0.99) for method B, and 0.93 (0.85–0.97) for method C. For exclusive breastfeeding among infants aged 0–5 months, estimated agreement with the validated district assignments was 0.43 (0.16–0.64) for method A, 0.99 (0.97–0.99) for method B, and 0.99 (0.97–0.99) for method C. These results indicate very strong agreement across methods B and C for each indicator. Method A was unfavorable for stunting among children under five and breastfeeding in children under 6 months.

### Estimate variability by district

**Fig. 3:**
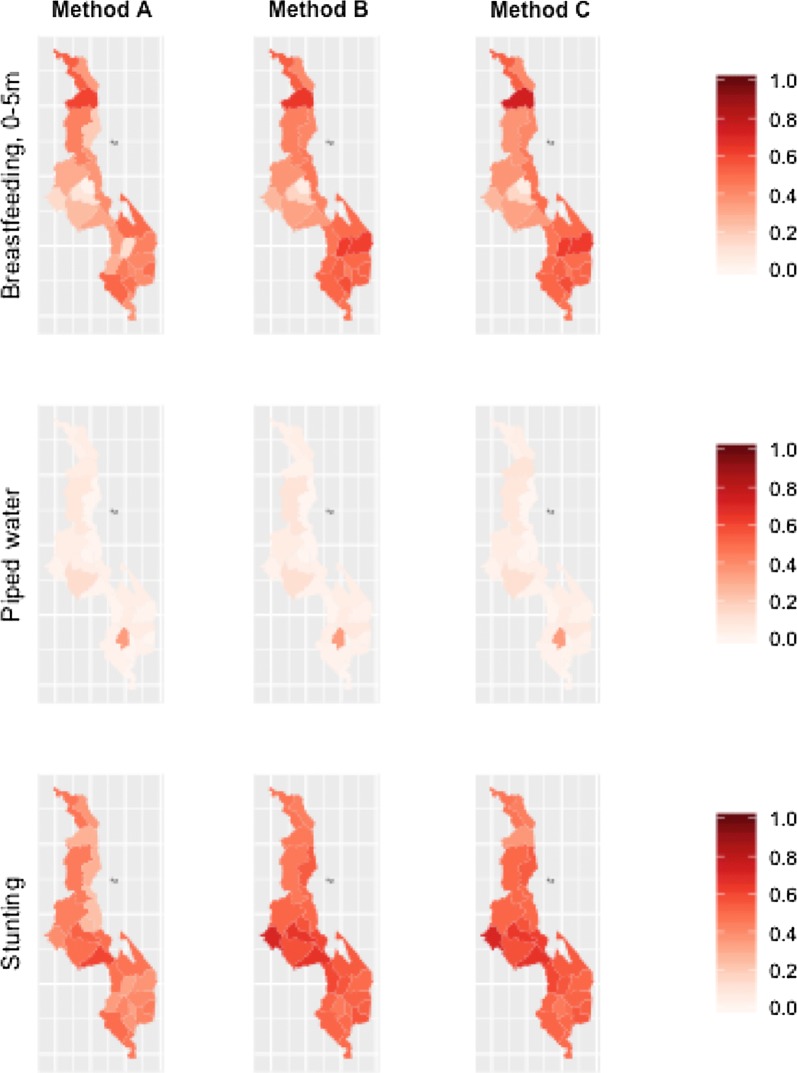
Estimated proportion coverage for districts by each method, in side-by-side maps, in 2000

We found broadly consistent estimates for a single district across different methods of district assignment. However, we did observe differences in coverage estimates depending on district. Coverage of piped water was lowest in Nsanje 0.00 (95% confidence not estimable), Machinga 0.00 (0.00, 0.01), and Ntchisi 0.00 (0.00, 0.01) and was highest in Blantyre 0.16 (0.09, 0.23) (Table 2). Moderate stunting was estimated to be the lowest in Karonga 0.37 (0.30, 0.45) and estimated to be the highest in Dedza 0.61 (0.53, 0.68) (Table 3). Exclusive breastfeeding in under 6 months ranged from 0.02 to 0.38 in Balaka, to 0.62 to 0.86 in Mulanje (Table 4). All three indicators showed real, actual variation between districts. Coverage levels are mapped for these indicators using method C and estimates that resulted using validated districts in Fig. 3.

When examining the district-level trend in coverage estimates between 2000 and 2004, there are apparent differences between district assignment methods for all coverages considered, especially for moderate stunting and exclusive breastfeeding. The estimated district-level trend was most different for the % with piped water in Rumphi district, ranging from a 1% decrease using method B and a 9% decrease using method C, and in Karonga district, ranging from a 6% increase with method A to a 12% increase with method C. For estimating the percent of children under five with moderate stunting, there were more extreme differences between district assignment methods, especially for Method A. In Mchinji district, method A estimated a 10% increase in stunting, while methods B and C estimated a 14% decrease. For estimating the percent of infants 0–5 months who are exclusively breastfed, estimated trends between 2000 and 2004 varied considerably. In Balaka district, method A estimated a 4% decrease in exclusive breast feeding, while methods B and C estimated a 41% decrease.

## Discussion

**Fig. 4:**
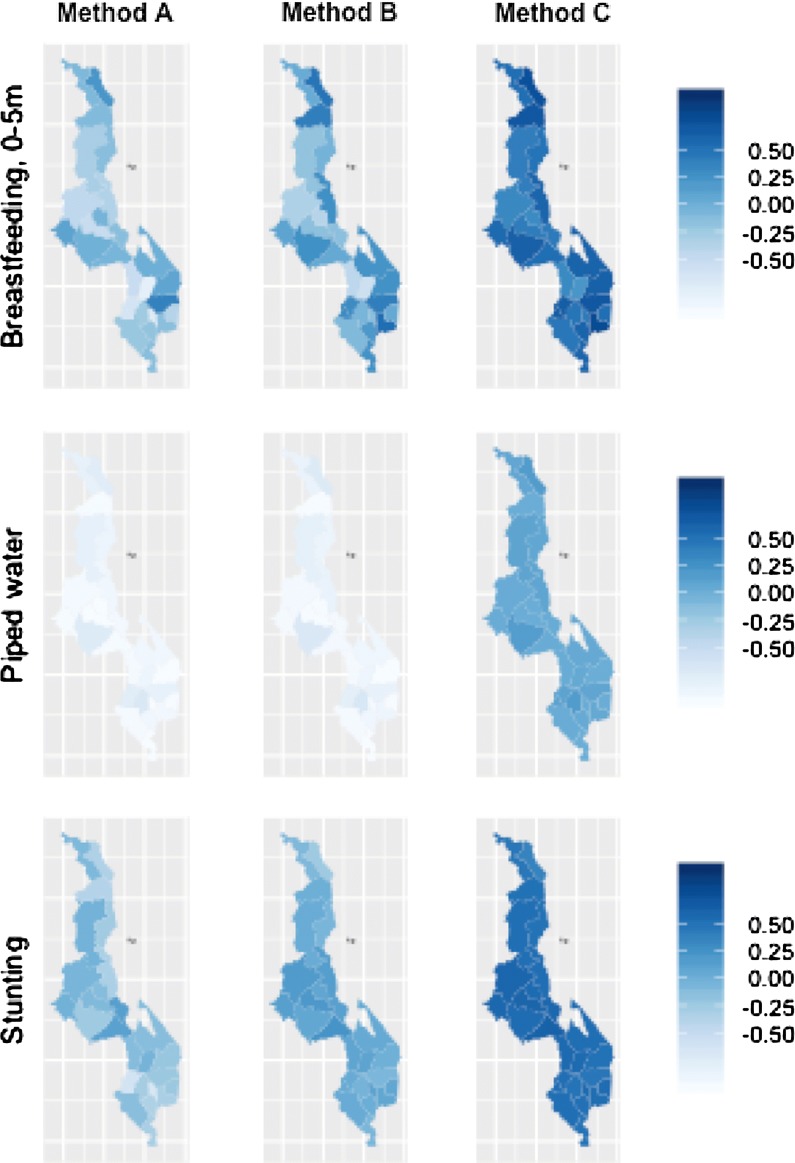
Estimated proportion coverage for districts by each method, in side-by-side maps, in 2004

We developed three methods to estimate where survey clusters of DHS surveys originated. Using each method, we estimated three coverage indicators which are commonly used in health system planning and program evaluation. We then compared the district-level estimates, confidence intervals, and agreement that came from each of the methods to the values from validated district locations in a single survey year, and compared estimated district-level trends over time between two surveys (Fig. 4).

Where we had validated district locations for comparison, we found that simulating possible district of origin, which is required for method A and method B, is not an improvement on using the geomasked coordinates directly. Methods A and B require more computational processing and analytical capacity than method C, which is simpler to implement and disseminate in low resources settings.

**Fig. 5:**
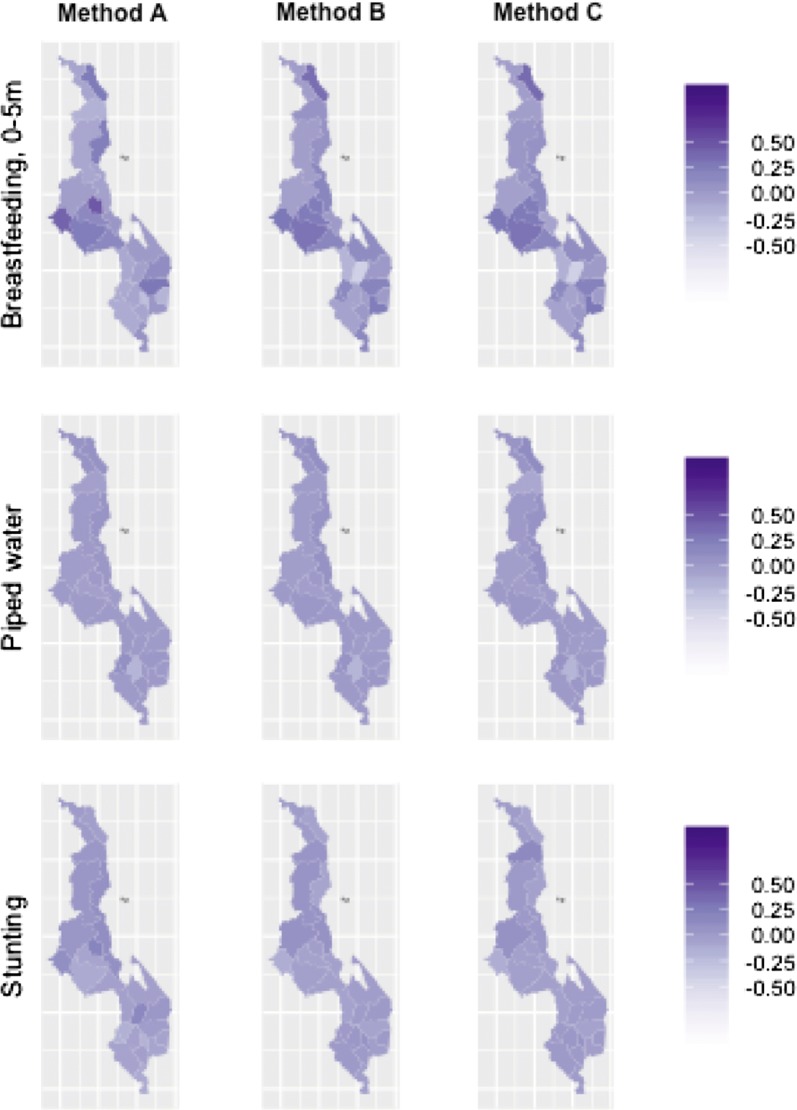
Estimated proportion coverage change by district, from 2000 to 2004, in side-by-side maps

**Fig. 6:**
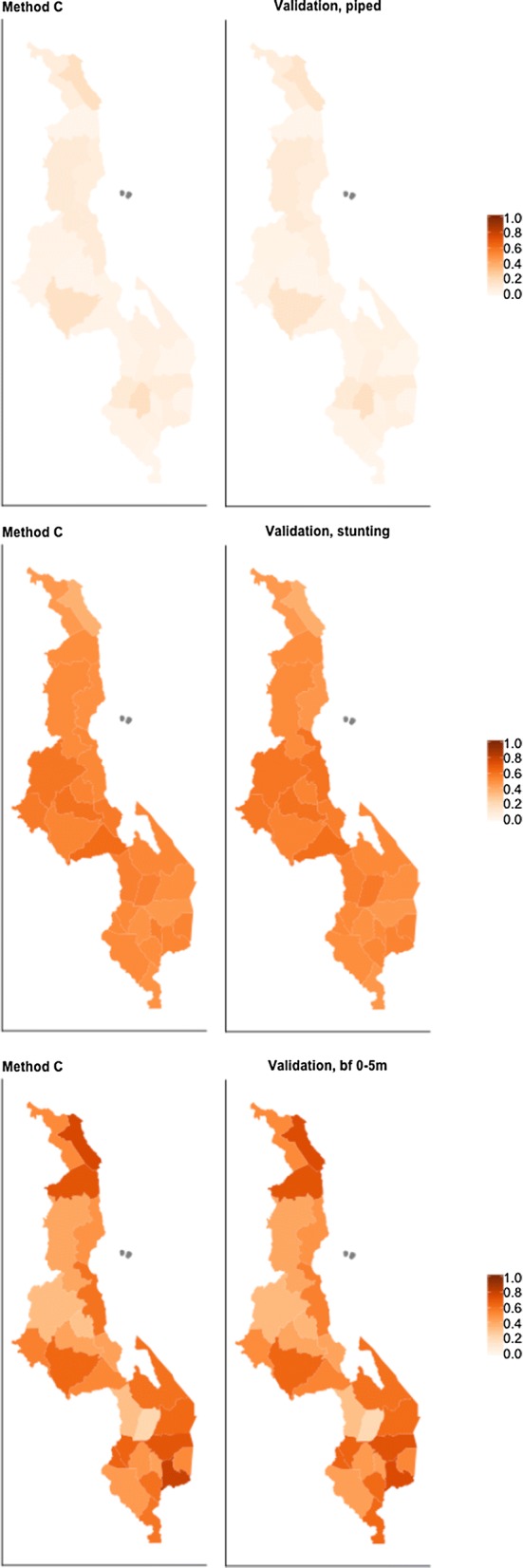
Estimated proportion coverage for districts by method C and validation method, in side-by-side maps, in 2004

We found that many survey clusters in the Malawi 2004 survey could be confirmed to have been geomasked within original district borders, where no district boundary was within the geomasking limit. Finding multiple possible districts of origin, as for methods A and B becomes irrelevant for surveys in 2009 or later, when DHS amended their geomasking process so that survey clusters are geomasked within district borders. Still we expect that these methods will be useful for estimating trends in survey data where an initial survey was conducted prior to 2009. We saw considerable variability in the trends in district-level estimates between these methods of district assignment, so the choice between methods would generally result in meaningful differences in estimated district-level trends. Although we were not able to estimate validated district-level trends over the period 2000–2004, we recommend using trend estimates using method C based on the high agreement between method C and validated district estimates in 2004 and the high availability of this method (Figs. 5, 6).

There are inherent limitations to using complex surveys such as the DHS that are designed for representation at the national or regional level for describing smaller areas such as districts. Household surveys are undertaken with great care and expense so that the best quality information is collected with pre-specified precision due to sampling variability. Optimal DHS sample size requires a trade-off between the resources available and the desired survey precision [23]. None of the methods considered allow that ideal and planned precision be maintained for district level estimation, where information is necessarily less enriched. In these instances, how much actual data is in each district may be of interest, since there is potential to have none, one, or few, survey clusters when these methods are used. For example, exclusive breastfeeding in neonates may result in uninformative estimates for many districts due to too few survey clusters. If sample sizes are too small, users may choose to group districts together or to consider alternate indicators.

These methods can be applied to countries whose DHSs sampled the population to be representative of regions or provinces, or an administrative level larger than districts, however, results may vary depending on the size and representation of each survey. There are 116 DHS surveys in 53 countries that collected GPS location of survey clusters prior to 2009 that also potentially geomasked survey cluster locations across district boundaries, representing many potential analyses of district-level estimates and the trends over time in these settings. We expect these methods to be useful in some but not all of these surveys, depending primarily on the precision of the district-level estimates, where less precise estimates require more caution in making assumptions about sensitivity to geomasked locations.

The intention for districts assigned using these methods is to estimate coverage, and the precision of coverage, for district populations. District assignments using these methods cannot necessarily be extrapolated when geomasked locations are being used for other purposes, such as estimating distances between households and health facilities. However, district assignments offer information about districts, even where survey design is not specific to the sampling of districts. Similarly, DHSs offer much information about subpopulations such as young infants, even though sampling design is not based on the size of those subpopulations.

Another limitation of our methods is that we have not included the district population in any of our potential assignment methods, which may improve the usability of our partial district assignment method A or the most probably district for method B. Incorporating population would require reliable district population estimates at the same time of survey, which may not be available for some surveys. We expect, however, that it is unlikely either methods A or B would be significantly improved here beyond the high agreement between method C and the validated district estimates.

## Conclusions

We can use geomasked geolocations from DHSs to describe coverage in districts in LMICs, nearly as if the DHS had provided the validated district of surveyed households. Comparing districts assigned this way to the validated districts, we found coverage estimates and confidence intervals that result from each method are effectively the same as the coverage estimates and confidence intervals that result from the validated assignments.

District data is necessary to better implement health programs, as well as to identify gaps in data where more information is needed. It is possible, with additional research, that these methods could be carried beyond descriptive analyses, and incorporated into hypothesis testing, predictive modeling, and statistical comparisons. Modeled and imputed data can be examined against direct evidence from each district to identify districts without data, where modelling and imputing remain necessary. We advocate ongoing investment in obtaining high-quality MNCH&N data in hard-to-reach subnational areas in LMICs. In the meantime, we recommend that governments, policy makers, implementers, and evaluators access district data for planning, implementing, and evaluating MNCH&N programs in LMICs.

## Supplementary information

**Additional file 1: Table S1–S3.** Household piped water, moderate stunting, and exclusive breastfeeding point estimates and 95% CIs, by district and method, in 2000 and 2004.

## Data Availability

The analysis supporting the conclusions of this article is available through a Zenodo DOI. 10.5281/zenodo.2634515.
