# Supplementary material for: Obtaining district-level health estimates using geographically masked location from Demographic and Health Survey data
Source: Int J Health Geogr. 2020 Feb 10;19:2. doi: 10.1186/s12942-020-0198-4 (PMC7011502; doi:10.1186/s12942-020-0198-4)
Supplement: Supplementary file 1 — Additional file 1: Table S1–S3. Household piped water, moderate stunting, and exclusive breastfeeding point estimates and 95% CIs, by district and method, in 2000 and 2004. [file 12942_2020_198_MOESM1_ESM.docx]

**Table S1a. 2000 household piped water point estimate and 95% CI, by district and method. Nsanje surveyed no households which reported having piped water; no confidence interval is reported.**

|  | **Method A** | | **Method B** | | **Method C** | |
| --- | --- | --- | --- | --- | --- | --- |
|  | **est** | **95% CI** | **est** | **95% CI** | **est** | **95% CI** |
| Balaka | .05 | [0.02, 0.08] | 0.06 | [0.02, 0.09] | 0.06 | [0.02, 0.09] |
| Blantyre | .34 | [0.22, 0.47] | 0.35 | [0.23, 0.47] | 0.35 | [0.23, 0.47] |
| Chikwawa | .02 | [0.00, 0.04] | 0.02 | [0.00, 0.04] | 0.02 | [0.00, 0.04] |
| Chiradzulu | .00 | [0.00, 0.01] | 0.00 |  | 0 | [0.00, 0.01] |
| Chitipa | .02 | [0.00, 0.06] | 0.02 | [0.00, 0.06] | 0.02 | [0.00, 0.06] |
| Dedza | .01 | [0.00, 0.03] | 0.01 | [0.00, 0.03] | 0.01 | [0.00, 0.03] |
| Dowa | .04 | [0.00, 0.12] | 0.04 | [0.00, 0.10] | 0.04 | [0.00, 0.1] |
| Karonga | .05 | [0.01, 0.08] | 0.04 | [0.01, 0.07] | 0.04 | [0.01, 0.07] |
| Kasungu | .04 | [0.01, 0.08] | 0.05 | [0.01, 0.09] | 0.05 | [0.01, 0.09] |
| Lilongwe | .13 | [0.05, 0.22] | 0.13 | [0.05, 0.2] | 0.13 | [0.05, 0.2] |
| Machinga | .01 | [0.00, 0.02] | 0.02 | [0.00, 0.04] | 0.02 | [0.00, 0.04] |
| Mangochi | .04 | [0.01, 0.07] | 0.04 | [0.01, 0.07] | 0.04 | [0.01, 0.07] |
| Mchinji | .01 | [0.00, 0.03] | 0.02 | [0.00, 0.05] | 0.02 | [0.00, 0.05] |
| Mulanje | .02 | [0.01, 0.04] | 0.02 | [0.01, 0.04] | 0.02 | [0.01, 0.04] |
| Mwanza | .02 | [0.00, 0.06] | 0.03 | [0.00, 0.10] | 0.03 | [0.00, 0.1] |
| Mzimba | .09 | [0.01, 0.16] | 0.10 | [0.04, 0.16] | 0.08 | [0.02, 0.14] |
| Nkhata Bay | .00 | [0.00, 0.01] | 0 |  | 0 |  |
| Nkhotakota | .01 | [0.00, 0.01] | 0.00 | [0.00, 0.01] | 0 | [0.00, 0.01] |
| Nsanje | 0 |  | 0.00 |  | 0 |  |
| Ntcheu | .01 | [0.00, 0.02] | 0.01 | [0.00, 0.02] | 0.01 | [0.00, 0.02] |
| Ntchisi | 0 |  | 0 |  | 0 |  |
| Phalombe | 0 |  | 0.02 | [0.00, 0.05] | 0.02 | [0.00, 0.05] |
| Rumphi | .05 | [0.01, 0.09] | 0.02 | [0.00, 0.06] | 0.1 | [0.02, 0.19] |
| Salima | .03 | [0.01, 0.05] | 0.03 | [0.01, 0.04] | 0.04 | [0.01, 0.06] |
| Thyolo | .01 | [0.00, 0.01] | 0.02 | [0.00, 0.03] | 0.01 | [0.00, 0.03] |
| Zomba | .05 | [0.00, 0.11] | 0.08 | [0.00, 0.14] | 0.08 | [0.01, 0.15] |

**Table S1b. Household piped water point estimate and 95% CI, by district and method, in 2004. Nsanje surveyed no households which reported having piped water; no confidence interval is reported.**

|  | **Method A** | | **Method B** | | **Method C** | | **Validation** | |
| --- | --- | --- | --- | --- | --- | --- | --- | --- |
|  | **est** | **95% CI** | **est** | **95% CI** | **est** | **95% CI** | **est** | **95% CI** |
| Balaka | 0.05 | [0.00, 0.13] | 0.04 | [0.00, 0.12] | 0.04 | [0.00, 0.12] | 0.05 | [0.00, 0.13] |
| Blantyre | 0.15 | [0.08, 0.22] | 0.16 | [0.09, 0.23] | 0.16 | [0.09, 0.23] | 0.16 | [0.09, 0.23] |
| Chikwawa | 0.02 | [0.00, 0.05] | 0.02 | [0.00, 0.05] | 0.02 | [0.00, 0.05] | 0.02 | [0.00, 0.05] |
| Chiradzulu | 0.00 | [0.00, 0.00] | 0.02 | [0.00, 0.05] | 0.02 | [0.00, 0.05] | 0.02 | [0.00, 0.05] |
| Chitipa | 0.08 | [0.00, 0.2] | 0.07 | [0.00, 0.17] | 0.07 | [0.00, 0.17] | 0.07 | [0.00, 0.17] |
| Dedza | 0.01 | [0.00, 0.03] | 0.02 | [0.00, 0.05] | 0.02 | [0.00, 0.05] | 0.02 | [0.00, 0.06] |
| Dowa | 0.00 |  | 0.01 | [0.00, 0.02] | 0.01 | [0.00, 0.02] | 0.01 | [0.00, 0.02] |
| Karonga | 0.11 | [0.00, 0.29] | 0.14 | [0.00, 0.34] | 0.16 | [0.00, 0.44] | 0.14 | [0.00, 0.34] |
| Kasungu | 0.02 | [0.00, 0.04] | 0.02 | [0.00, 0.05] | 0.02 | [0.00, 0.05] | 0.02 | [0.00, 0.04] |
| Lilongwe | 0.13 | [0.03, 0.22] | 0.15 | [0.04, 0.25] | 0.15 | [0.04, 0.25] | 0.14 | [0.04, 0.23] |
| Machinga | 0.00 | [0.00, 0.00] | 0.00 | [0.00, 0.00] | 0.00 | [0.00, 0.00] | 0.00 | [0.00, 0.01] |
| Mangochi | 0.03 | [0.00, 0.06] | 0.04 | [0.00, 0.07] | 0.04 | [0.00, 0.08] | 0.04 | [0.00, 0.07] |
| Mchinji | 0.01 | [0.00, 0.03] | 0.01 | [0.00, 0.03] | 0.01 | [0.00, 0.03] | 0.01 | [0.00, 0.02] |
| Mulanje | 0.03 | [0.01, 0.05] | 0.04 | [0.02, 0.07] | 0.05 | [0.02, 0.08] | 0.04 | [0.01, 0.07] |
| Mwanza | 0.11 | [0.00, 0.29] | 0.09 | [0.00, 0.25] | 0.09 | [0.00, 0.25] | 0.09 | [0.00, 0.25] |
| Mzimba | 0.08 | [0.01, 0.16] | 0.09 | [0.02, 0.16] | 0.09 | [0.02, 0.16] | 0.09 | [0.02, 0.16] |
| Nkhata Bay | 0.05 | [0.00, 0.15] | 0.06 | [0.00, 0.15] | 0.06 | [0.00, 0.17] | 0.06 | [0.00, 0.15] |
| Nkhotakota | 0.03 | [0.00, 0.09] | 0.07 | [0.00, 0.19] | 0.08 | [0.00, 0.22] | 0.05 | [0.00, 0.15] |
| Nsanje | 0.00 |  | 0.00 |  | 0 |  | 0 |  |
| Ntcheu | 0.02 | [0.00, 0.04] | 0.02 | [0.00, 0.04] | 0.02 | [0.00, 0.05] | 0.02 | [0.00, 0.04] |
| Ntchisi | 0.00 |  | 0.01 | [0.00, 0.01] | 0.01 | [0.00, 0.01] | 0.00 | [0.00, 0.01] |
| Phalombe | 0.01 | [0.00, 0.02] | 0.01 | [0.00, 0.02] | 0.01 | [0.00, 0.02] | 0.01 | [0.00, 0.02] |
| Rumphi | 0.01 | [0.00, 0.03] | 0.01 | [0.00, 0.02] | 0.01 | [0.00, 0.02] | 0.01 | [0.00, 0.02] |
| Salima | 0.02 | [0.00, 0.04] | 0.02 | [0.00, 0.03] | 0.02 | [0.00, 0.04] | 0.02 | [0.00, 0.03] |
| Thyolo | 0.03 | [0.01, 0.06] | 0.04 | [0.01, 0.06] | 0.04 | [0.01, 0.06] | 0.03 | [0.01, 0.06] |
| Zomba | 0.08 | [0.01, 0.14] | 0.08 | [0.01, 0.14] | 0.08 | [0.01, 0.14] | 0.08 | [0.01, 0.14] |

**Table S2a. Moderate stunting point estimates and 95% CI, by district and method, in 2000**

|  | **Method A** | | **Method B** | | **Method C** | |
| --- | --- | --- | --- | --- | --- | --- |
|  | **est** | **95% CI** | **est** | **95% CI** | **est** | **95% CI** |
| Balaka | 0.33 | [0.14, 0.51] | 0.56 | [0.45, 0.67] | 0.56 | [0.45, 0.67] |
| Blantyre | 0.41 | [0.35, 0.48] | 0.45 | [0.4, 0.51] | 0.45 | [0.4, 0.51] |
| Chikwawa | 0.49 | [0.43, 0.55] | 0.51 | [0.47, 0.56] | 0.51 | [0.47, 0.56] |
| Chiradzulu | 0.33 | [0.17, 0.49] | 0.49 | [0.41, 0.56] | 0.48 | [0.41, 0.56] |
| Chitipa | 0.47 | [0.38, 0.55] | 0.47 | [0.38, 0.55] | 0.47 | [0.38, 0.55] |
| Dedza | 0.60 | [0.51, 0.69] | 0.66 | [0.6, 0.71] | 0.66 | [0.60, 0.71] |
| Dowa | 0.52 | [0.39, 0.66] | 0.65 | [0.61, 0.69] | 0.64 | [0.59, 0.69] |
| Karonga | 0.36 | [0.29, 0.43] | 0.44 | [0.4, 0.47] | 0.44 | [0.4, 0.47] |
| Kasungu | 0.44 | [0.36, 0.52] | 0.51 | [0.46, 0.56] | 0.51 | [0.46, 0.56] |
| Lilongwe | 0.48 | [0.4, 0.57] | 0.57 | [0.51, 0.63] | 0.57 | [0.51, 0.63] |
| Machinga | 0.37 | [0.25, 0.48] | 0.49 | [0.44, 0.54] | 0.50 | [0.45, 0.56] |
| Mangochi | 0.47 | [0.37, 0.56] | 0.54 | [0.48, 0.61] | 0.54 | [0.48, 0.61] |
| Mchinji | 0.39 | [0.16, 0.62] | 0.71 | [0.64, 0.77] | 0.71 | [0.64, 0.77] |
| Mulanje | 0.42 | [0.35, 0.49] | 0.56 | [0.52, 0.6] | 0.56 | [0.52, 0.59] |
| Mwanza | 0.35 | [0.14, 0.55] | 0.52 | [0.36, 0.67] | 0.52 | [0.36, 0.67] |
| Mzimba | 0.45 | [0.36, 0.54] | 0.46 | [0.36, 0.55] | 0.50 | [0.44, 0.57] |
| Nkhata Bay | 0.28 | [0.03, 0.54] | 0.54 | [0.46, 0.62] | 0.54 | [0.46, 0.62] |
| Nkhotakota | 0.23 | [0.09, 0.37] | 0.50 | [0.42, 0.58] | 0.50 | [0.42, 0.58] |
| Nsanje | 0.46 | [0.37, 0.55] | 0.50 | [0.43, 0.56] | 0.50 | [0.43, 0.56] |
| Ntcheu | 0.48 | [0.35, 0.6] | 0.60 | [0.53, 0.66] | 0.60 | [0.53, 0.66] |
| Ntchisi | 0.25 | [0.1, 0.41] | 0.57 | [0.55, 0.59] | 0.57 | [0.55, 0.59] |
| Phalombe | 0.45 | [0.31, 0.58] | 0.53 | [0.44, 0.62] | 0.53 | [0.44, 0.62] |
| Rumphi | 0.28 | [0.13, 0.43] | 0.46 | [0.37, 0.55] | 0.36 | [0.26, 0.46] |
| Salima | 0.48 | [0.38, 0.59] | 0.60 | [0.53, 0.67] | 0.62 | [0.57, 0.67] |
| Thyolo | 0.36 | [0.27, 0.46] | 0.50 | [0.45, 0.56] | 0.50 | [0.45, 0.56] |
| Zomba | 0.40 | [0.33, 0.48] | 0.52 | [0.47, 0.57] | 0.52 | [0.47, 0.57] |

**Table S2b. Moderate stunting point estimates and 95% CI, by district and method, in 2004**

|  | **Method A** | | **Method B** | | **Method C** | | **Validation** | |
| --- | --- | --- | --- | --- | --- | --- | --- | --- |
|  | **est** | **95% CI** | **est** | **95% CI** | **est** | **95% CI** | **est** | **95% CI** |
| Balaka | 0.48 | [0.37, 0.58] | 0.54 | [0.49, 0.6] | 0.54 | [0.49, 0.6] | 0.57 | [0.52, 0.62] |
| Blantyre | 0.35 | [0.28, 0.43] | 0.48 | [0.41, 0.54] | 0.48 | [0.41, 0.54] | 0.49 | [0.42, 0.55] |
| Chikwawa | 0.43 | [0.36, 0.5] | 0.51 | [0.46, 0.57] | 0.51 | [0.46, 0.57] | 0.49 | [0.44, 0.55] |
| Chiradzulu | 0.16 | [0.00, 0.31] | 0.55 | [0.45, 0.64] | 0.55 | [0.45, 0.64] | 0.51 | [0.4, 0.62] |
| Chitipa | 0.46 | [0.39, 0.52] | 0.45 | [0.4, 0.51] | 0.45 | [0.4, 0.51] | 0.45 | [0.4, 0.51] |
| Dedza | 0.56 | [0.46, 0.65] | 0.62 | [0.54, 0.69] | 0.62 | [0.54, 0.69] | 0.61 | [0.53, 0.68] |
| Dowa | 0.45 | [0.31, 0.59] | 0.6 | [0.54, 0.65] | 0.59 | [0.54, 0.65] | 0.59 | [0.54, 0.65] |
| Karonga | 0.32 | [0.23, 0.41] | 0.37 | [0.3, 0.45] | 0.36 | [0.27, 0.46] | 0.37 | [0.3, 0.45] |
| Kasungu | 0.47 | [0.39, 0.55] | 0.58 | [0.53, 0.63] | 0.58 | [0.53, 0.63] | 0.58 | [0.54, 0.62] |
| Lilongwe | 0.37 | [0.29, 0.45] | 0.53 | [0.45, 0.61] | 0.53 | [0.45, 0.61] | 0.54 | [0.46, 0.61] |
| Machinga | 0.39 | [0.31, 0.47] | 0.49 | [0.43, 0.55] | 0.49 | [0.43, 0.55] | 0.49 | [0.43, 0.55] |
| Mangochi | 0.43 | [0.36, 0.51] | 0.52 | [0.47, 0.57] | 0.52 | [0.47, 0.57] | 0.51 | [0.47, 0.56] |
| Mchinji | 0.49 | [0.39, 0.58] | 0.57 | [0.5, 0.64] | 0.57 | [0.5, 0.64] | 0.58 | [0.49, 0.67] |
| Mulanje | 0.34 | [0.24, 0.45] | 0.53 | [0.47, 0.6] | 0.53 | [0.46, 0.6] | 0.54 | [0.47, 0.6] |
| Mwanza | 0.19 | [0.03, 0.35] | 0.51 | [0.39, 0.64] | 0.51 | [0.39, 0.64] | 0.51 | [0.39, 0.64] |
| Mzimba | 0.48 | [0.42, 0.54] | 0.51 | [0.47, 0.55] | 0.51 | [0.47, 0.55] | 0.51 | [0.47, 0.55] |
| Nkhata Bay | 0.36 | [0.21, 0.52] | 0.47 | [0.4, 0.54] | 0.49 | [0.42, 0.55] | 0.47 | [0.4, 0.54] |
| Nkhotakota | 0.33 | [0.2, 0.47] | 0.57 | [0.44, 0.69] | 0.52 | [0.41, 0.63] | 0.59 | [0.49, 0.69] |
| Nsanje | 0.34 | [0.18, 0.51] | 0.46 | [0.37, 0.56] | 0.48 | [0.37, 0.58] | 0.46 | [0.37, 0.56] |
| Ntcheu | 0.44 | [0.33, 0.54] | 0.54 | [0.48, 0.6] | 0.54 | [0.48, 0.61] | 0.54 | [0.48, 0.6] |
| Ntchisi | 0.44 | [0.31, 0.56] | 0.54 | [0.45, 0.63] | 0.54 | [0.45, 0.63] | 0.53 | [0.45, 0.62] |
| Phalombe | 0.38 | [0.24, 0.53] | 0.53 | [0.4, 0.66] | 0.53 | [0.41, 0.66] | 0.53 | [0.4, 0.66] |
| Rumphi | 0.31 | [0.15, 0.47] | 0.49 | [0.41, 0.58] | 0.49 | [0.41, 0.58] | 0.49 | [0.41, 0.58] |
| Salima | 0.57 | [0.53, 0.6] | 0.56 | [0.53, 0.59] | 0.57 | [0.53, 0.6] | 0.55 | [0.52, 0.59] |
| Thyolo | 0.33 | [0.24, 0.42] | 0.5 | [0.45, 0.55] | 0.5 | [0.45, 0.55] | 0.52 | [0.47, 0.57] |
| Zomba | 0.38 | [0.3, 0.47] | 0.46 | [0.4, 0.52] | 0.46 | [0.4, 0.52] | 0.46 | [0.39, 0.52] |

**Table S3a. Exclusive breastfeeding point estimates and 95% CI, by district and method, in 2000**

|  | **Method A** | | **Method B** | | **Method C** | |
| --- | --- | --- | --- | --- | --- | --- |
|  | **est** | **95% CI** | **est** | **95% CI** | **est** | **95% CI** |
| Balaka | 0.13 | [0.01, 0.26] | 0.62 | [0.38, 0.87] | 0.62 | [0.38, 0.87] |
| Blantyre | 0.45 | [0.34, 0.56] | 0.53 | [0.43, 0.64] | 0.53 | [0.43, 0.64] |
| Chikwawa | 0.51 | [0.34, 0.68] | 0.51 | [0.37, 0.66] | 0.51 | [0.37, 0.66] |
| Chiradzulu | 0.4 | [0.12, 0.68] | 0.45 | [0.28, 0.62] | 0.44 | [0.27, 0.62] |
| Chitipa | 0.53 | [0.18, 0.89] | 0.53 | [0.18, 0.89] | 0.53 | [0.18, 0.89] |
| Dedza | 0.32 | [0.19, 0.45] | 0.35 | [0.23, 0.47] | 0.35 | [0.23, 0.47] |
| Dowa | 0.1 | [0.01, 0.19] | 0.16 | [0.07, 0.26] | 0.16 | [0.07, 0.25] |
| Karonga | 0.35 | [0.22, 0.47] | 0.4 | [0.27, 0.53] | 0.4 | [0.27, 0.53] |
| Kasungu | 0.3 | [0.18, 0.42] | 0.37 | [0.24, 0.5] | 0.37 | [0.24, 0.5] |
| Lilongwe | 0.25 | [0.13, 0.36] | 0.33 | [0.2, 0.45] | 0.33 | [0.2, 0.45] |
| Machinga | 0.43 | [0.27, 0.59] | 0.62 | [0.49, 0.75] | 0.63 | [0.5, 0.76] |
| Mangochi | 0.49 | [0.31, 0.68] | 0.49 | [0.32, 0.67] | 0.49 | [0.32, 0.67] |
| Mchinji | 0.15 | [0.04, 0.26] | 0.26 | [0.12, 0.41] | 0.26 | [0.12, 0.41] |
| Mulanje | 0.4 | [0.3, 0.5] | 0.52 | [0.4, 0.65] | 0.51 | [0.39, 0.64] |
| Mwanza | 0.28 | [0.01, 0.55] | 0.5 | [0.27, 0.73] | 0.5 | [0.27, 0.73] |
| Mzimba | 0.43 | [0.29, 0.58] | 0.43 | [0.25, 0.62] | 0.37 | [0.21, 0.53] |
| Nkhata Bay | 0.2 | [0, 0.41] | 0.41 | [0.21, 0.62] | 0.41 | [0.21, 0.62] |
| Nkhotakota | 0.36 | [0.04, 0.69] | 0.46 | [0.1, 0.83] | 0.46 | [0.1, 0.83] |
| Nsanje | 0.37 | [0.08, 0.66] | 0.47 | [0.11, 0.83] | 0.47 | [0.11, 0.83] |
| Ntcheu | 0.34 | [0.21, 0.48] | 0.47 | [0.34, 0.59] | 0.47 | [0.34, 0.59] |
| Ntchisi | 0.02 | [0, 0.05] | 0.06 | [0, 0.16] | 0.06 | [0, 0.16] |
| Phalombe | 0.49 | [0.21, 0.77] | 0.52 | [0.31, 0.74] | 0.52 | [0.31, 0.74] |
| Rumphi | 0.61 | [0.48, 0.74] | 0.64 | [0.52, 0.76] | 0.73 | [0.56, 0.9] |
| Salima | 0.4 | [0.23, 0.56] | 0.42 | [0.25, 0.6] | 0.49 | [0.33, 0.65] |
| Thyolo | 0.48 | [0.35, 0.61] | 0.58 | [0.47, 0.69] | 0.58 | [0.47, 0.69] |
| Zomba | 0.41 | [0.27, 0.55] | 0.51 | [0.4, 0.61] | 0.49 | [0.39, 0.6] |

**Table S3b. Exclusive breastfeeding point estimates and 95% CI, by district and method, in 2004**

|  | **Method A** | | **Method B** | | **Method C** | | **Validation** | |
| --- | --- | --- | --- | --- | --- | --- | --- | --- |
|  | **est** | **95% CI** | **est** | **95% CI** | **est** | **95% CI** | **est** | **95% CI** |
| Balaka | 0.09 | [0.00, 0.19] | 0.21 | [0.05, 0.37] | 0.21 | [0.05, 0.37] | 0.20 | [0.02, 0.38] |
| Blantyre | 0.37 | [0.23, 0.51] | 0.47 | [0.32, 0.62] | 0.47 | [0.32, 0.62] | 0.45 | [0.30, 0.60] |
| Chikwawa | 0.39 | [0.25, 0.52] | 0.45 | [0.29, 0.61] | 0.45 | [0.29, 0.61] | 0.43 | [0.28, 0.59] |
| Chiradzulu | 0.13 | [0.00, 0.31] | 0.43 | [0.24, 0.62] | 0.43 | [0.24, 0.62] | 0.43 | [0.21, 0.65] |
| Chitipa | 0.45 | [0.21, 0.7] | 0.51 | [0.27, 0.75] | 0.51 | [0.27, 0.75] | 0.51 | [0.27, 0.75] |
| Dedza | 0.47 | [0.33, 0.61] | 0.52 | [0.4, 0.65] | 0.52 | [0.40, 0.65] | 0.53 | [0.40, 0.67] |
| Dowa | 0.24 | [0.09, 0.4] | 0.42 | [0.3, 0.54] | 0.42 | [0.30, 0.54] | 0.41 | [0.29, 0.53] |
| Karonga | 0.6 | [0.36, 0.85] | 0.74 | [0.51, 0.96] | 0.75 | [0.52, 0.99] | 0.74 | [0.51, 0.96] |
| Kasungu | 0.27 | [0.17, 0.37] | 0.32 | [0.22, 0.43] | 0.32 | [0.22, 0.43] | 0.33 | [0.23, 0.43] |
| Lilongwe | 0.49 | [0.37, 0.6] | 0.63 | [0.52, 0.74] | 0.63 | [0.52, 0.74] | 0.63 | [0.53, 0.73] |
| Machinga | 0.54 | [0.41, 0.67] | 0.62 | [0.54, 0.71] | 0.62 | [0.54, 0.71] | 0.62 | [0.53, 0.70] |
| Mangochi | 0.5 | [0.34, 0.65] | 0.62 | [0.49, 0.74] | 0.60 | [0.46, 0.73] | 0.60 | [0.48, 0.72] |
| Mchinji | 0.55 | [0.3, 0.79] | 0.55 | [0.36, 0.73] | 0.55 | [0.36, 0.73] | 0.50 | [0.32, 0.69] |
| Mulanje | 0.42 | [0.23, 0.62] | 0.76 | [0.64, 0.88] | 0.79 | [0.67, 0.90] | 0.74 | [0.62, 0.86] |
| Mwanza | 0.18 | [0.00, 0.39] | 0.65 | [0.38, 0.91] | 0.65 | [0.38, 0.91] | 0.65 | [0.38, 0.91] |
| Mzimba | 0.34 | [0.23, 0.45] | 0.4 | [0.28, 0.52] | 0.40 | [0.28, 0.52] | 0.41 | [0.28, 0.53] |
| Nkhata Bay | 0.43 | [0.22, 0.64] | 0.48 | [0.3, 0.66] | 0.48 | [0.30, 0.66] | 0.48 | [0.30, 0.66] |
| Nkhotakota | 0.32 | [0.11, 0.52] | 0.63 | [0.39, 0.88] | 0.59 | [0.34, 0.84] | 0.55 | [0.31, 0.78] |
| Nsanje | 0.43 | [0.12, 0.74] | 0.62 | [0.32, 0.93] | 0.58 | [0.25, 0.91] | 0.62 | [0.32, 0.93] |
| Ntcheu | 0.24 | [0.09, 0.39] | 0.28 | [0.14, 0.42] | 0.31 | [0.15, 0.47] | 0.31 | [0.16, 0.45] |
| Ntchisi | 0.48 | [0.36, 0.59] | 0.3 | [0.13, 0.47] | 0.30 | [0.13, 0.47] | 0.35 | [0.18, 0.51] |
| Phalombe | 0.3 | [0.1, 0.51] | 0.52 | [0.41, 0.63] | 0.51 | [0.40, 0.62] | 0.52 | [0.41, 0.63] |
| Rumphi | 0.45 | [0.08, 0.81] | 0.7 | [0.54, 0.86] | 0.70 | [0.54, 0.86] | 0.70 | [0.54, 0.86] |
| Salima | 0.43 | [0.26, 0.6] | 0.42 | [0.28, 0.56] | 0.43 | [0.28, 0.58] | 0.42 | [0.27, 0.56] |
| Thyolo | 0.4 | [0.23, 0.57] | 0.6 | [0.47, 0.73] | 0.60 | [0.47, 0.73] | 0.62 | [0.50, 0.74] |
| Zomba | 0.69 | [0.53, 0.85] | 0.7 | [0.58, 0.82] | 0.70 | [0.58, 0.82] | 0.71 | [0.59, 0.83] |
